# Medical education at crossroads: Recommendations from a national study in Pakistan

**DOI:** 10.12669/pjms.343.15377

**Published:** 2018

**Authors:** Zarrin Seema Siddiqui

**Affiliations:** 1 Dr. Zarrin S. Siddiqui, PhD. MD Education Unit, The University of Western Australia, Perth, Australia

## INTRODUCTION

Pakistan Medical and Dental Council (PM&DC) is the statutory regulatory and registration authority for medical and dental education in Pakistan.^1^ However earlier this year, the Supereme Court of Pakistan disbanded the Council and an interim committee has been set to manage the affairs.^2^ The Committee is currently trying to restructure the medical and dental programs offered in Pakistan. It is important at this critical time, that the voices of medical graduates who are the major stakeholders are heard to get an insight into the issues from their perspective. In this article, recommendations from a major national study are presented to investigate career intentions of medical graduates and to identify the influencing factors. The study used a sequential mixed method approach which included six focus group discussions and a questionnaire survey to which four hundred and seventy-nine medical graduates from twenty-four medical schools responded.

These recommendations may assist the policy planners in considering and planning appropriate interventions, according to the stage of training to improve the retention of the medical workforce in Pakistan.

Following are the twenty recommendations:

### 1. Offer career guidance at secondary school level

The schools and higher degree institutions should use different forums to provide guidance to high school leavers and their families about the options available for further education other than medicine. Summer schools could provide students with some awareness about different fields in higher education which may help in decision making.

Due to the remote geographical locations in Pakistan, it may not be possible to arrange face to face sessions in all parts of the country hence the use of both electronic and print media could be considered.

### 2. Portray a realistic image of the medical profession

There is an expectation by the prospective students that they will be able to treat patients in an unsupervised practice immediately upon graduation. In addition, there is a perception about the medical profession believing the graduates are able to earn a high income soon after graduation and support their families.

Dispel the myths surrounding the medical profession to both prospective students and their families by providing information about the opportunities available, and explain that graduation is just the beginning phase in the life of a dcotor.

### 3. Reconsider the entry criteria to medical course

The entry requirements of medical course are changing worldwide, with more medical schools opting for a graduate entry. In Pakistan, this discussion has not even started and perhaps it is time to review the entry criteria. One option may be to have two years of under graduate pre-clinical teaching, followed by an examination that leads to admission into a postgraduate medical program which may be structured around four years. For those who are unsuccessful, an option to complete another year of education with a Bachelor’s degree in Basic Sciences or Health Sciences could be offered. This would provide prospective medical students with an opportunity to think before they enter a course. It would also provide them an exit option in case their circumstances change, or if they can not gain entry into medicine or if they do not want to pursue medicine as a career any more.

### 4. Provision of a conducive learning environment

The most encouraging thing that has happenned in recent times is that the PM&DC has provided medical schools more flexibility in planning and delivering the curriculum. This was acknowledged by the students, yet they also highlighted the need to restructure clinical rotations as they mainly rely on academic staff (both in clinical and basic medical sciences) who have been appointed by the the Public Service Commission in public institutions. Often these staff do not take teaching of students seriously and they are not accountable to the medical college administration.

Another concern raised by the graduates in this study was “part time medical college” where faculty though appointed full-time are hardly available after 2 pm. At the same time, during morning hours academics have other responsibilities like attending meetings and rounds.

A learning environment is dependent on adequate planning and resources, both physical and human. A learning environment that encourages autonomy facilitates feelings of competence among students and may also increase their interest in a specialty.^3^ In this study, the responsibility towards management of patients was rated highly by students as it encouraged them to learn. This activity needs to be properly supervised.

The composition of the faculty staff is important but so is the provision of appropriate faculty development activities as these affect the effective supervision of the graduates to provide the adequate patient care.^4^ Medical education units are now present in all universities and can play a key role in enhancing the skills of the faculty with support from the institutions.

### 5. Provision of electives

Summer/winter schools and electives in different specialties could be made available to students so that they could explore different areas of interest. The electives could also be offered in non-traditional disciplines like; medical education, bioethics and research intensive subjects to attract the graduates.

### 6. Ensure adequate student support

While there is a general trend toward specialization, it is important to maintain a balance between general practice and specializations to strengthen the health care system and to identify where there is a shortage of specialists so that medical graduates can make informed decisions based on this information.

There is evidence that medical schools can assist students in making career decisions as reported in a study that compared medical students in 1993 to medical students in 1996 in a medical course and found that there was an increase in the feelings of preparedness and confidence.^5^

The time table during the medical course could be scheduled to include protected time for students to meet specialists and receive career guidance from them. It is also up to the specialists to provide a realistic picture of their professions.

In collaboration with different organsiations, career fairs could be introduced or College of Physicians and Surgeons Pakistan could organise one career fair annually with seminars to appraise prospective graduates of the opportunities that are available to them and how to make the best use of them.

### 7. Introduce interprofessional learning experiences

The role of paramedical staff emerged very strongly as a deterrent for the medical graduates. While this area needs more investigation, some form of carefully planned interprofessional learning experiences may help to narrow the gap between different professions.

### 8. Improve security and safety measures

The most commonly identified concern was security and safety within the working environment. To address this at higher level, policy planners need to take measures that ensure the safety of all the personnel involved in providing healthcare services.

### 9. Structure training programs at all levels

Clear outcomes for the different levels of training along with appropriate learning experiences should be provided to the students. There was no difference found among teaching activites for house officers and postgraduate trainees, hence a number of teaching activities were not found to be useful by house officers. The house job is currently only structured around time and not the content. There should be clearer guidelines as to what is expected during and at the end of house job.

### 10.Provide effective assessment and feedback

The assessment modes used in undergraduate and postgraduate training could be reviewed and updated to include more contemporary methods of assessment such as programmatic assessment,^6^ incorporating workplace based assessment with more opportunities for feedback.

### 11.Introduce a national examination

One of the problems identified in the current study was the difficulty experienced by house officers in gaining entry to postgraduate training programs which require the medical graduates to be assessed on their basic knowledge of medical subjects. Graduates feel that they have to study for the entry examination which may sometimes take a year or so. One option to improve the situation could be to have two national examinations, one at the end of the first two years of the medical course and another close to graduation. The top ten to twenty percent of students could be offerred direct entry into Fellowship or Masters program as well as the results may be used for indigenous and overseas schoalrships.

Another use of this examination could be to use the results for appointments into the public and private hospital sector. This would alleviate the multiple examinations that graduates currently have to take for different boards. A third way to use the examination results could be for benchmarking medical institutions and identify areas for improvement as part of internal evaluation.

### 12.Increase Salary/Funding/ Research assistantships

The government in Pakistan is already taking steps to provide trainees with an adequate stipend at both the Federal and the Provincial levels. Salaries for both house job and postgraduate levels have been revised and increased.

There may be more opportunities for house officers and trainees to work as research assitants in projects to earn some income and to also further their careers at the same time. This could be implemented by government and training institutions through allocation of specific funds for research activities. The trainees in the current study raised the issue that while there is a requirement for them to submit a research dissertation, there is no provision of funding to conduct the research. The Higher Education Commission has introduced a number of research grants schemes since its inception in 2001 and many of these are directed towards funding of PhD students. There is a need that some research funding may be exclusively allocated for use by postgraduate trainees in Fellowship training.

Pharmaceutical companies who already provide funding to some specialists to travel overseas could be asked to invest a similar amount in to research funding for the postgraduate trainees.

### 13.Develop user friendly policies and procedures

Women are in the majority in Pakistan in terms of entry in to medical schools and will soon be in the the majority of workforce.Gender specific policies that promote gender equality are important and it should be ensured that no graduate is disadvantaged on the basis of gender.

Key organisations such as Pakistan Medical and Dental Council, College of Physicians and Surgeons Pakistan, Pakistan Medical Asociation need to develop policies and guidelines for implementation by medical institutions. The committees that develop these policies should also ideally include membership from women, trainees and community representatives who can provide broader insight into issues that may be culturally and gender specific. The policies should address provision of leave during house job and postgraduate training which do not disadvantage anyone.

In this study, issues were raised of bullying, mistreatment and harassment. In order to address these issues, policies and confidential mechansims need to be introduced and enforced in medical institutions. There should also be a centralised facility where graduates/students/trainees could confidentially report these issues and where appropriate action against those responsible could be initiated. This is a very difficult step to take as the appointments of different health professionals are governed by federal and provincial health authorities. Instigating any inquiry into lodged actions is difficult. Private institutions have a similar problem however accreditation of superviors and institutions can be affected if the number of complaints against an institution/department/supervisor is increasing and can be substantiated with evidence.

### 14.Introduce return to practice programs

It is inevitable that some graduates will need to take some time off from the profession for before different reasons. This may result in a gap that may range from one to few years. In a country with already crippling resources in terms of medical manpower, bridging programs may be worthwhile as they might be able to bring back those who have been away from the profession. Another option could be to provide part-time options so that the gradautes are not lost to other professions.

### 15.Provide more flexibility to postgraduate trainees

Flexibility within medical course and postgraduate training program is also required.

Modular postgraduate programs could be introduced. One private university is already experimenting with this by providing a flexible post graduate training option while College of Physicians and Surgeons Pakistan has introduced a Membership in Family Medicine, where medical graduates can sit in the Membership examination based on experience in the field. The recognition of prior learning in the fields where graduates are already working could also be applied in different specialties.

Medical institutions need to take a good look at the postgraduate training process to consider whether they could be adapted to accommodate those trainees who choose to specialise, but would prefer a part-time pathway in order to accommodate personal obligations.

Career satisfaction is inversely correlated with burnout and depression. If a person is provided with an option to complete a residency on a full-time or part-time basis, it could be more satisfying to the graduate. They may even experience more support from their families.

A second aspect to offer more flexibility would be to structure training programs so that if a trainee exits at certain points, he/she can still get a qualification, so the time spent in training is not wasted. For example, a taining program could offer trainees a choice of receiving a postgraduate certificate, diploma or a degree, depending on the time that they spent in training with or without examination. These qualifications could be standardised across different institutes for the purpose of employment or, if at a later stage, the gradauate wants to recommence the training.

### 16.Ensure appropriate representation on committees

Though already discussed in earlier recommendation, there is a need to form committees with wider representation from academics at varying stages of their careers as well as students and community representatives with adequate timelines to complete the given tasks. The recommendations from these committees can assist policy makers to make regulations that could streamline the induction of medical graduates in the workforce. If successful, this model can apply to other members of health professional teams.

### 17.External review of the two major organisations responsible for medical education in Pakistan

Although the two major organisations PM&DC and CPSP were established as an autonomous organisations, one of the findings in the current study was that medical graduates raised concerns about the monopoly of these organisations in regulating medical education in the country. An external review of the two organisations could be conducted to identify areas for improvement.

### 18.Professional organisations review their roles in providing career advice to medical students and graduates

Almost every specialty has its own professional society which is involved in supporting the medical graduates. The current study found that medical graduates do not feel adequately supported by their own institutions or other organsiations. This leaves room for professional organisations/societies to intervene and to assume responsibility for providing some sort of career advice, mentoring and education of medical graduates about their specialty.

### 19.Establish consortia

Currently a number of medical colleges are affiliated with a university. The universities could join hands together to form a consortium which offers mobility to postgraduate trainees and supervisors can support each other. These alliances could also be used to develop joint degree programs and collaborative projects in different disciplines.

### 20.Initiate research in medical education and provide workforce estimates

Careful workforce needs analysis is required to predict the number requirements of specialists required in Pakistan. The Association of American Medical Colleges (AAMC) and its member schools follow students’ career intentions with the data obtained from the annual Medical School Graduation Questionnaire (GQ). Analyses may include review of current data, special topic evaluation, and trend analysis. A similar system could be introduced in Pakistan as PM&DC requires each medical student to register at the time of entering the medical course. PM&DC can release annual data about how many graduates have been registered and what their locations of practice are to provide a realsitic estimate of number of doctors serving in different areas and specialties.

## CONCLUSION

A medical graduate has to make several decisions upon graduation. Firstly, whether to practice or not, secondly whether or not to specialise and finally which specialty to choose for postgraduation. These decisions are made at graduation, or soon after, and are the result of a combination of circumstances. In this article, twenty recommendations have been presented to facilitate the decision making process of the medical graduates in their career intentions and to improve their retention in the workforce.

A number of issues for further research have emerged from this study. This could provide a broader view of the trends in the career intentions of medical graduates as well as, how these trends will affect the workforce, the educational process and the quality of medical care to patients.
